# ChromID: A Protocol for Mapping Protein Chromatin Interactions in Living Cells

**DOI:** 10.21769/BioProtoc.5807

**Published:** 2026-09-05

**Authors:** Richard Cardoso da Silva, Douwe ten Bulte, Tuncay Baubec

**Affiliations:** Genome Biology and Epigenetics, Institute of Biodynamics and Biocomplexity, Department of Biology, Utrecht University, Utrecht, The Netherlands

**Keywords:** ChromID, Biotin proximity labeling, Engineered chromatin readers, Chromatin modification interactome, LC-MS/MS proteomics, Histone post-translational modifications

## Abstract

Chromatin modifications regulate genome function by recruiting proteins that control transcription, genome organization, and DNA repair. Identifying the proteins associated with specific chromatin modifications is therefore essential for understanding how these regulatory processes operate. Traditional approaches, including chromatin immunoprecipitation and affinity purification coupled to mass spectrometry, have uncovered many chromatin-associated proteins. However, they often rely on crosslinking and chromatin fragmentation, which can disrupt native chromatin architecture and limit the detection of transient interactions. Here, we describe a proximity-labeling protocol for identifying the chromatin-dependent protein interactome associated with specific chromatin marks, termed ChromID. ChromID uses engineered chromatin readers (eCRs) fused to a promiscuous biotin ligase, which labels proteins in the immediate vicinity of the targeted chromatin mark. The protocol includes in vivo biotin labeling, nuclear extract preparation, streptavidin-based enrichment, and tryptic digestion for downstream LC-MS/MS analysis. The protocol has been validated across multiple cell types and chromatin contexts and can be extended to other chromatin-associated proteins, providing a versatile approach to profile chromatin-associated proteomes within their native cellular environment.

Key features

• Maps proteins associated with different chromatin modifications in living cells using engineered chromatin readers fused to TurboID, BASU, or other promiscuous biotin ligases.

• Preserves native chromatin organization and captures transient chromatin-associated interactions that are often lost during conventional affinity purification workflows.

• Validated across multiple chromatin contexts, including histone modifications, DNA methylation, transcription factors, RNA polymerase II, and DNA damage-associated chromatin states.

• Applicable to diverse cell types and organisms and adaptable to other chromatin-associated proteins, including transcription factors and chromatin regulators.

## Graphical overview


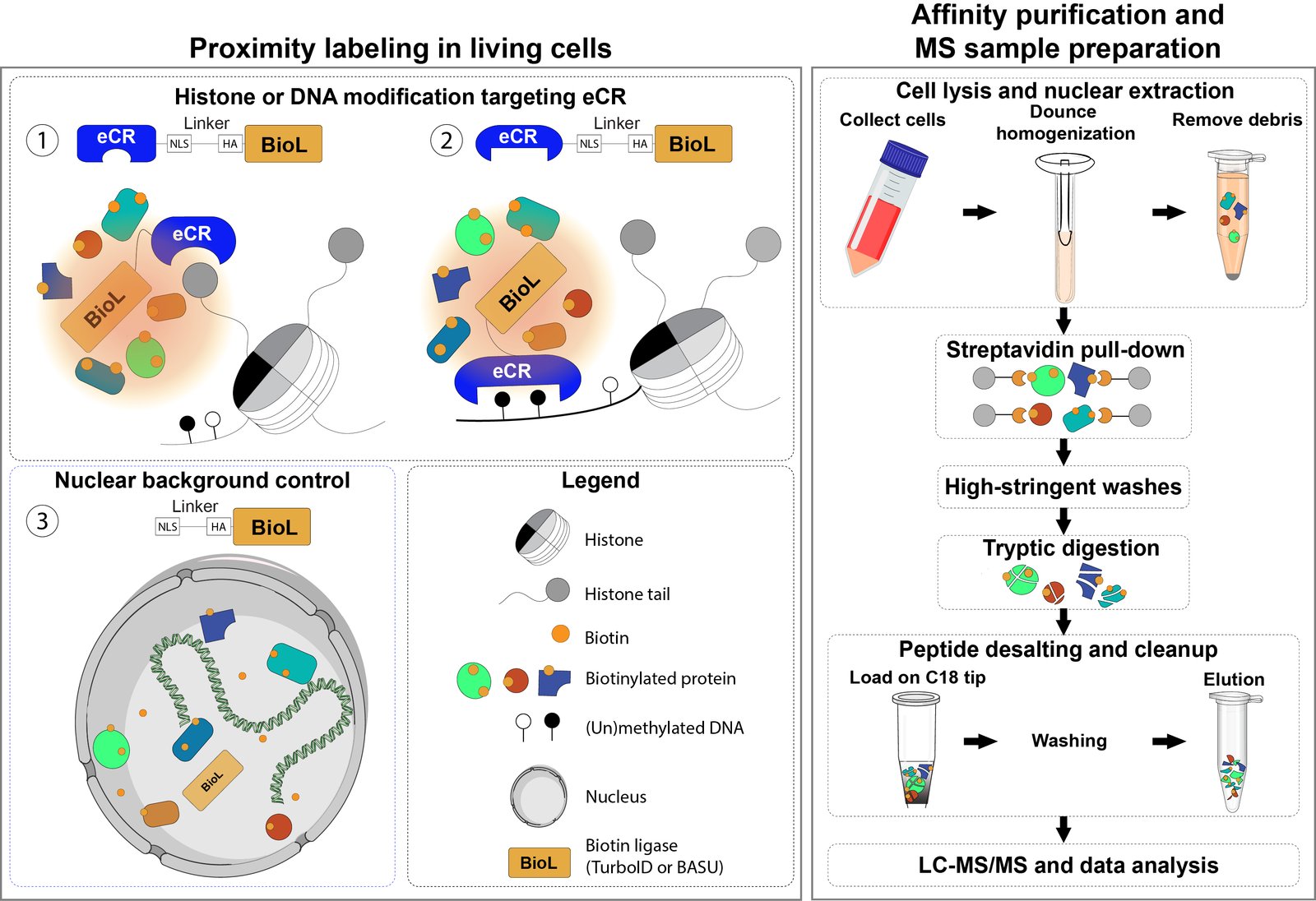


**Graphical overview of the ChromID protocol, where engineered chromatin readers (eCRs), fused to promiscuous biotin ligases, target specific chromatin modifications, including histone and DNA modifications, and label proximal proteins for identification by liquid chromatography–tandem mass spectrometry (LC-MS/MS).** The figure is intended as a schematic representation of the workflow and does not depict a specific eCR. Representative eCRs are summarized in Supplementary Table 1. Nuclear BioL, without the reader, is used as the background control.

## Background

Chromatin modifications, including histone post-translational modifications and DNA methylation, are central regulators of genome function and cellular identity. These modifications serve as molecular signals that are recognized by chromatin-associated effector proteins, often referred to as chromatin readers. These readers interpret local chromatin states to coordinate diverse biological processes, including RNA transcription, DNA replication, and repair [1]. Mapping the protein networks associated with specific chromatin modifications is therefore critical for understanding how epigenetic states are established, maintained, and remodeled during development and disease.

Several biochemical and proteomic strategies have been developed to characterize chromatin-associated protein complexes, including co-immunoprecipitation, affinity purification coupled to mass spectrometry, histone peptide and nucleosome pull-downs, and chromatin immunoprecipitation-based methods (reviewed in [2]). These approaches have provided important insights, but they share key limitations. They generally do not preserve the surrounding chromatin environment needed to capture context-dependent binding and often rely on chromatin fragmentation, crosslinking, affinity purification, or in vitro reconstitution. Such steps can disrupt native chromatin organization and cause the loss of transient interactions. Furthermore, antibody-based methods can be constrained by potential epitope blocking and nonspecific binding.

Proximity biotinylation has emerged as a powerful alternative for mapping protein interaction networks directly in living cells [3]. In this approach, promiscuous biotin ligases such as *Bacillus subtilis* BirA (BASU) or TurboID are fused to a bait protein, allowing covalent biotinylation of nearby proteins once biotin is added. Because labeling happens in intact cells before lysis, proximity biotinylation captures protein interactions in their native cellular environment, including the transient and low-affinity interactions often lost during conventional purification. Covalent biotin labeling also allows stringent washing protocols, resulting in reduced nonspecific background and improved reproducibility.

ChromID builds on this strategy by using the binding affinity of naturally occurring chromatin reader domains as modular building blocks to generate engineered chromatin readers (eCRs). Depending on the selected reader domain, eCRs can be engineered to recognize specific histone post-translational modifications and DNA modifications.

When fused to a promiscuous biotin ligase such as BASU or TurboID and stably expressed in cells, ChromID enables the identification of chromatin-associated protein networks directly in living cells. For example, DNA modification-targeting eCRs incorporate methyl-CpG-binding domains that selectively recognize 5-methylcytosine (5mC), enabling the characterization of proteins associated with methylated DNA in living cells. ChromID has also been used to profile protein networks associated with H3K4me3, H3K9me3, H3K27me3 and bivalent promoters marked by H3K4me3, and H3K27me3 [4]. The modular design of eCRs has also enabled mapping of protein networks associated with γH2AX-marked DNA damage sites [5] and actively elongating RNA polymerase II phosphorylated at serine 2 [6]. Representative examples of histone- and DNA modification-targeting eCRs and their applications are summarized in Supplementary Table 1.

Here, we describe a detailed ChromID sample preparation workflow covering biotin labeling, nuclear extract preparation, streptavidin affinity purification under stringent conditions, and tryptic digestion to produce high-quality peptides ready for LC-MS/MS analysis (Graphical overview). Like other proximity-labeling approaches, ChromID relies on the stability, proper chromatin localization, and efficient activity of the eCR-biotin ligase fusion protein, all of which should be experimentally validated prior to proteomic analysis. The workflow has been validated across multiple biological contexts, including mouse embryonic stem cells (mESCs), neural progenitor cells (NPCs), and human osteosarcoma cells (U2OS) in different cell cycle stages and under multiple genotoxic conditions. Furthermore, this protocol can be applied to any chromatin-associated protein of interest, including transcription factors [7] and other chromatin regulators, extending its applicability across diverse applications in chromatin biology, epigenetics, and beyond.

## Materials and reagents

**Biological materials**

Cell line of interest stably expressing engineered chromatin readers (eCRs) fused to BASU or TurboID. For example:

1. Mouse embryonic stem cells stably expressing eCRs fused to BASU or TurboID. Detailed protocols for the generation and validation of these cell lines are available under the Validation of protocol section and in [4,6].

2. Mouse embryonic stem cells stably expressing BASU or TurboID carrying a nuclear localization signal (NLS). Detailed protocols for the generation and validation of these cell lines are available in [4,6]. This cell line is used for background normalization.

3. U2OS stably expressing eCRs fused to TurboID. Detailed protocols for the generation and validation of these cell lines are available in [5].

4. U2OS stably expressing TurboID carrying an NLS. Detailed protocols for the generation and validation of these cell lines are available in [5]. This cell line is used for background normalization.

*Note: All plasmids used to generate these cell lines are available at Addgene:* *https://www.addgene.org/Tuncay_Baubec/* .

**Reagents**

1. Acetonitrile (ACN), LC-MS grade (Supelco, catalog number: 1.00029.1000)

2. Benzonase (Merck, catalog number: 71206)

3. Chloroacetamide (CAA) (Sigma-Aldrich, catalog number: C0267)

4. D-Biotin (Thermo Fisher Scientific, catalog number: B20656)

5. Dulbecco’s phosphate-buffered saline (DPBS) solution (VWR, catalog number: 392-0434)

6. DTT solution 1 M (Merck, catalog number: 43816-10ML)

7. EDTA disodium salt (EDTA) (Sigma-Aldrich, catalog number: ED2SS)

8. Formic acid (FA), MS grade (Merck, catalog number: 5.33002.0050)

9. Glycerol (Merck, catalog number: 356350)

10. HEPES (Sigma-Aldrich, CAS number: 7365-45-9)

11. Hydrochloric acid (HCl) (Supelco, catalog number: 1.00317)

12. IGEPAL CA-630 (IGEPAL) (Sigma-Aldrich, CAS number: 9002-93-1)

13. Lithium chloride (LiCl) (Sigma-Aldrich, CAS number: 7447-41-8)

14. Magnesium chloride hexahydrate (MgCl_2_) (Sigma-Aldrich, catalog number: M2670)

15. Methanol, LC-MS grade (Thermo Scientific, catalog number: 47192)

16. Potassium chloride (KCl) (Sigma-Aldrich, CAS number: 7447-40-7)

17. Protease inhibitor complex, cOmplete, EDTA-free (PIC) (Roche, catalog number: 0505648900)

18. Sodium chloride (NaCl) (Sigma-Aldrich, CAS number: 7647-14-5)

19. Sodium deoxycholate (NaDOC) (Sigma-Aldrich, CAS number: 302-95-4)

20. Sodium dodecyl sulfate (SDS) (Sigma-Aldrich, CAS number: 151-21-3)

21. Sodium hydroxide (NaOH) (Honeywell, CAS number: 1310-73-2)

22. Trifluoroacetic acid (TFA), MS-grade (Biosolve, catalog number: 202341)

23. Tris-base (Sigma-Aldrich, CAS number: 77-86-1)

24. Triton X-100 (Sigma-Aldrich, CAS number: 1003339794)

25. Trypsin, MS grade (Promega, catalog number: V5113)

26. Urea (Cytiva, CAS number: 17-1319-01)

27. Water, LC-MS compatible (Merck, catalog number: 1153331000)

**Solutions**

1. 500 mM HEPES pH 7.5 stock (see Recipes)

2. 1 M Tris pH 7.5 stock (see Recipes)

3. 1 M Tris pH 8.0 stock (see Recipes)

4. 1 M KCl stock (see Recipes)

5. 5 M NaCl stock (see Recipes)

6. 500 mM EDTA stock (see Recipes)

7. 500 mM MgCl_2_ stock (see Recipes)

8. 1 M LiCl stock (see Recipes)

9. 100× protease inhibitor complex (PIC) stock (see Recipes)

10. 10% SDS stock (see Recipes)

11. 5% NaDOC stock (see Recipes)

12. 500 mM chloroacetamide stock (see Recipes)

13. 0.1 μg/μL trypsin stock (see Recipes)

14. Tris-EDTA (TE) pH 8 buffer (see Recipes)

15. 2% SDS in TE stock (see Recipes)

16. 10% TFA solution (see Recipes)

17. 10% IGEPAL solution (see Recipes)

18. Nuclear extraction buffer 1 (NEB1) (see Recipes)

19. Nuclear extraction buffer 2, no-salt (NEB2-NS) (see Recipes)

20. Nuclear extraction buffer 2, 450 mM-salt (NEB2-450) (see Recipes)

21. Immuno-precipitation buffer (IPB) (see Recipes)

22. Deoxycholate buffer (DOC) (see Recipes)

23. High-salt buffer (HSB) (see Recipes)

24. RIPA buffer (see Recipes)

25. Urea elution buffer (see Recipes)

26. Spin Tip buffer A (see Recipes)

27. Spin Tip buffer B (see Recipes)

**Recipes**

**1. 500 mM HEPES pH 7.5 stock**

To approximately 400 mL of Milli-Q water (MQ), add 59.58 g of HEPES and stir until dissolved. Adjust pH to 7.5 with NaOH, then bring to a final volume of 500 mL with MQ. Store at room temperature (RT) for ~1 year.

**2. 1 M Tris pH 7.5 stock**

To approximately 400 mL of MQ, add 60.57 g of Tris-base and stir until dissolved. Adjust pH to 7.5 with HCl, then bring to a final volume of 500 mL with MQ. Store at RT for ~1 year.

**3. 1 M Tris pH 8.0 stock**

To approximately 400 mL of MQ, add 60.57 g of Tris-base and stir until dissolved. Adjust pH to 8.0 with HCl, then bring to a final volume of 500 mL with MQ. Store at RT for ~1 year.

**4. 1 M KCl stock**

To approximately 400 mL of MQ, add 37.28 g of KCl and stir until dissolved. Bring to a final volume of 500 mL with MQ. Store at RT for ~1 year.

**5. 5 M NaCl stock**

To approximately 400 mL of MQ, add 146.10 g of NaCl and stir until dissolved. Bring to a final volume of 500 mL with MQ. Store at RT for ~1 year.

**6. 500 mM EDTA stock**

To approximately 400 mL of MQ, add 93.06 g of EDTA and stir until dissolved. Adjust pH to 8.0 with NaOH to improve the solubility of EDTA, then bring to a final volume of 500 mL with MQ. Store at RT for ~1 year.

**7. 500 mM MgCl_2_ stock**

To approximately 400 mL of MQ, add 50.83 g of MgCl_2_ and stir until dissolved. Bring to a final volume of 500 mL with MQ. Store at RT for ~1 year.

**8. 1 M LiCl stock**

To approximately 400 mL of MQ, add 21.20 g of LiCl and stir until dissolved. Bring to a final volume of 500 mL with MQ. Store at RT for ~1 year.

**9. 100× PIC stock**

Dissolve 10 tablets of protease inhibitor complex in 5 mL of MQ for a final 100× stock. Aliquot in 0.5 mL and store at -20 °C to increase lifespan (~1 year). Aliquots can be thawed 2–3 times.

**10. 10% SDS stock**

To approximately 80 mL of MQ, add 10.0 g of SDS and stir until dissolved. Then bring to a final volume of 100 mL with MQ. Store at RT for ~1 year. This solution might precipitate below RT.

**11. 5% NaDOC stock**

To approximately 40 mL of MQ, add 2.5 g of NaDOC and stir until dissolved. Adjust to 50 mL with MQ. Store at RT and protected from light. Lifespan ~1 year.

**12. 500 mM chloroacetamide stock**

To approximately 1.75 mL of MQ, add 93.5 mg of chloroacetamide and shake until dissolved. Adjust to 2 mL with MQ. Aliquot in 0.1 mL and store at -20 °C in the dark. Lifespan ~6 months. Avoid reusing thawed aliquots.

**13. 0.1 μg/μL trypsin stock**

Trypsin (V5113) contains two reagents: 0.5 μg/μL trypsin (V511C) and trypsin resuspension dilution buffer (V542A). Thaw both reagents from -80 °C. Dilute trypsin to 0.1 μg/μL with the resuspension buffer. Make 0.1 mL aliquots and store at -80 °C. Avoid freeze-thaw cycles; aliquots can be used ~3 times.

**14. TE pH 8 buffer**

Mix 5 mL of 1 M Tris stock with 1 mL of 500 mM EDTA, pH 8, and bring to a final volume of 500 mL with MQ. Store at RT for ~1 year.

**15. 2% SDS in TE stock**

To approximately 200 mL of TE pH 8.0, add 5.0 g of SDS and stir until dissolved. Gentle heating at 37 °C may help with dissolution. Bring to a final volume of 250 mL with TE pH 8.0. Store at RT for ~1 year. Might precipitate below RT. Before usage add 1 M DTT to a final concentration of 1 mM. This working solution can be used for 1 day.

**16. 10% TFA solution**

Dilute 200 μL of TFA with 1,800 μL of MQ. Prepare this stock solution fresh on the day of usage, as it is highly light-sensitive.

**17. 10% IGEPAL solution**

Dilute 1 mL of IGEPAL with 9 mL of MQ. Store the solution at RT for ~1 year.

**18. NEB1**

**Table:** 

Reagent	Final concentration	Quantity or volume
500 mM HEPES pH 7.5 stock	10 mM	5 mL
1 M KCl stock	10 mM	2.5 mL
500 mM EDTA stock	1 mM	500 μL
500 mM MgCl_2_ stock	1.5 mM	750 μL
1 M DTT	1 mM	Add fresh*
100× PIC stock	1×	Add fresh*
MQ Water	n/a	To 250 mL
Total	n/a	250 mL

*Note: Store stock solution (250 mL) without DTT and PIC at 4 °C for 1 year. On the day of use, freshly add DTT and the PIC to the final working concentration. Keep this working solution at 4 °C, which can be used for 1 day.*

**19. NEB2-NS**

**Table:** 

Reagent	Final concentration	Quantity or volume
500 mM HEPES pH 7.5 stock	20 mM	10 mL
Glycerol	20% (v/v)	50 mL
500 mM EDTA stock	0.2 mM	100 μL
500 mM MgCl_2_ stock	1.5 mM	750 μL
1 M DTT	1 mM	Add fresh*
100× PIC stock	1×	Add fresh*
MQ	n/a	To 250 mL
Total	n/a	250 mL

*Note: Store stock solution (250 mL) without DTT and PIC at 4 °C for 1 year. On the day of use, freshly add DTT and PIC to the final working concentration. Keep this working solution at 4 °C, which can be used for 1 day.*

**20. NEB2-450**

**Table:** 

Reagent	Final concentration	Quantity or volume
500 mM HEPES pH 7.5 stock	20 mM	10 mL
Glycerol	20% (v/v)	50 mL
500 mM EDTA stock	0.2 mM	100 μL
500 mM MgCl_2_ stock	1.5 mM	750 μL
5 M NaCl stock	450 mM	22.5 mL
1 M DTT	1 mM	Add fresh*
100× PIC stock	1×	Add fresh*
MQ	n/a	To 250 mL
Total	n/a	250 mL

*Note: Store stock solution (250 mL) without DTT and PIC at 4 °C for 1 year. On the day of use, freshly add DTT and PIC to the final working concentration. Keep this working solution at 4 °C, which can be used for 1 day.*

**21. IPB**

**Table:** 

Reagent	Final concentration	Quantity or volume
500 mM HEPES pH 7.5 stock	20 mM	10 mL
Glycerol	20% (v/v)	50 mL
IGEPAL	0.3% (v/v)	750 μL
500 mM EDTA stock	0.2 mM	100 μL
500 mM MgCl_2_ stock	1.5 mM	750 μL
5 M NaCl stock	150 mM	7.5 mL
1 M DTT	1 mM	Add fresh*
100× PIC stock	1×	Add fresh*
MQ	n/a	To 250 mL
Total	n/a	250 mL

*Note: Store stock solution (250 mL) without DTT and PIC at 4 °C for 1 year. On the day of use, freshly add DTT and PIC to the final working concentration. Keep this working solution at 4 °C, which can be used for 1 day.*

**22. DOC**

**Table:** 

Reagent	Final concentration	Quantity or volume
1 M LiCl stock	250 mM	50 mL
1 M Tris pH 8.0 stock	10 mM	2.5 mL
IGEPAL	0.5% (v/v)	1.250 mL
5% NaDOC stock	0.5% (w/v)	25 mL
500 mM EDTA stock	1 mM	100 μL
1 M DTT	1 mM	Add fresh*
100× PIC stock	1×	Add fresh*
MQ	n/a	To 250 mL
Total	n/a	250 mL

*Note: Store stock solution (250 mL) without DTT and PIC at 4 °C for 1 year in the dark. On the day of use, freshly add DTT and PIC to the final working concentration. Keep this working solution at 4 °C, which can be used for 1 day.*

**23. HSB**

**Table:** 

Reagent	Final concentration	Quantity or volume
500 mM HEPES pH 7.5 stock	50 mM	25 mL
500 mM EDTA stock	1 mM	500 μL
Triton X-100	1% (v/v)	2.5 mL
5% NaDOC stock	0.1% (w/v)	5 mL
10% SDS stock	0.1% (w/v)	2.5 mL
5 M NaCl stock	500 mM	25 mL
1 M DTT	1 mM	Add fresh*
100× PIC stock	1×	Add fresh*
MQ	n/a	To 250 mL
Total	n/a	250 mL

*Note: Store stock solution (250 mL) without DTT and PIC at 4 °C for 1 year in the dark. On the day of use, freshly add DTT and PIC to the final working concentration. Keep this working solution at 4 °C, which can be used for 1 day.*

**24. RIPA buffer**

**Table:** 

Reagent	Final concentration	Quantity or volume
1 M Tris pH 7.5 stock	25 mM	6.25 mL
5 M NaCl stock	150 mM	7.5 mL
IGEPAL	1% (v/v)	2.5 mL
5% NaDOC stock	0.1% (w/v)	5 mL
10% SDS stock	0.1% (w/v)	2.5 mL
1 M DTT	1 mM	Add fresh*
100× PIC stock	1×	Add fresh*
MQ	n/a	To 250 mL
Total	n/a	250 mL

*Note: Store stock solution (250 mL) without DTT and PIC at 4 °C for 1 year in the dark. On the day of use, freshly add DTT and PIC to the final working concentration. Keep this working solution at 4 °C, which can be used for 1 day.*

**25. Urea elution buffer**

**Table:** 

Reagent	Final concentration	Quantity or volume
1 M Tris pH 8.0 stock	100 mM	1 mL
Urea	2 M	1.2 g
1 M DTT	10 mM	100 μL
Total	n/a	10 mL

*Note: Prepare this solution fresh immediately before use. To minimize contamination, use urea dedicated exclusively to MS sample preparation.*

**26. Spin Tip buffer A**

**Table:** 

Reagent	Final concentration	Quantity or volume
MS-grade formic acid (100% v/v)	0.1% (v/v)	10 μL
Water, LC-MS compatible	n/a	9,990 μL
Total	n/a	10 mL

*Note: Prepare this solution fresh immediately before use.*

**27. Spin Tip buffer B**

**Table:** 

Reagent	Final concentration	Quantity or volume
MS-grade acetonitrile	80% (v/v)	8 mL
MS-grade formic acid	0.1% (v/v)	10 μL
Water, LC-MS compatible	n/a	1,990 μL
Total	n/a	10 mL

*Note: Prepare this solution fresh immediately before use.*

**Laboratory supplies**

1. Streptavidin Sepharose high-performance beads (Cytiva, catalog number: 17511301)

2. Safe-Lock tubes 1.5 mL (Eppendorf, catalog number: 0030120086)

3. Safe-Lock tubes 2 mL (Eppendorf, catalog number: 0030120094)

4. Conical tube 15 mL (Sarstedt, catalog number: 62.554.502)

5. Conical tube 50 mL (Sarstedt, catalog number: 62.547.254)

6. C18 Spin tips (Pierce, catalog number: 87784)

7. 20 μL pipette tips (Sarstedt, catalog number: 703.020.100)

8. 200 μL pipette tips (Sarstedt, catalog number: 703.030.100)

9. 1,250 μL pipette tips (Sarstedt, catalog number: 703.060.100)

10. Nitrile gloves (VWR, catalog number: 112-4196)

11. BD Microlance 26G needle (BD, catalog number: 303800)

12. pH indicator strips, MQuant (Supelco, catalog number: 1.09535.0001)

13. Combitips advanced, 0.5 mL (Eppendorf, catalog number: 0030089782)

14. Low protein binding tubes 1.5 mL (Thermo Scientific, catalog number: 90410)

15. Qubit Broad Range Protein assay (Thermo Scientific, catalog number: Q33211)

16. Cell scraper (VWR, catalog number: 734-2604)

17. Cell culture plates 15 cm (MLS, catalog number: Y93150)

18. α-Lamin-B1 antibody (Santa Cruz Biotechnology, catalog number: sc-6216)

19. High-sensitivity Streptavidin-HRP (Thermo Scientific, catalog number: 21130)

20. Streptavidin-Alexa Fluor 790 (Thermo Scientific, catalog number: S11378)

## Equipment

1. Eppendorf ThermoMixer C (Eppendorf, catalog number: 5382000015)

2. Centrifuge (Eppendorf, model: 5425 R)

3. Centrifuge (Eppendorf, model: 5810 R)

4. Multi-rotator (Grant instruments, model: PTR-35)

5. Ultra-low temperature freezer (Eppendorf, model: F570 series)

6. Vacuum centrifuge, Savant Speedvac (Thermo Scientific, model: DNA1300)

7. Research Plus pipettes (0.1–2.5 μL) (Eppendorf, catalog number: 3123000217)

8. Research Plus pipettes (2–20 μL) (Eppendorf, catalog number: 3123000292)

9. Research Plus pipettes (20–200 μL) (Eppendorf, catalog number: 3123000250)

10. Research Plus pipettes (100–1,000 μL) (Eppendorf, catalog number: 3123000268)

11. Qubit 3.0 Fluorometer (Thermo Scientific, catalog number: Q33216)

12. Kimble Dounce tissue grinder set (2 mL) (Sigma-Aldrich, catalog number: D8938-1SET)

13. Cell counter (Bio-Rad, model: TC20)

14. Cold room (4 °C) or refrigerator (Liebherr, model: GKv 6410 ProfiLine)

15. Tabletop vortexer (Scientific Industries, model: Vortex-Genie 2)

16. UHPLC (Thermo Scientific, model: UltiMate 3000 UHPLC system)

17. Mass spectrometer (Thermo Scientific, model: Orbitrap Exploris 480)

18. Milli-Q water purification system (Millipore, model: Integral 3)

## Software and datasets

*Note: All software listed is freely available.*

1. MaxQuant (Max Planck Institute of Biochemistry, version 1.6.2.6) includes the integrated Andromeda search engine; source: https://www.maxquant.org [8,9]

2. Perseus (Max Planck Institute of Biochemistry, version 1.6.14.0); source: https://www.maxquant.net/perseus [10]

3. PTXQC (open-source R package, version 1.0 or later), used for LC-MS/MS quality control; source: https://github.com/cbielow/PTXQC [11]

4. R (R Foundation for Statistical Computing, version 4.3.0 or later), open source (GPL license), used for quality control and data visualization; source: https://www.r-project.org

5. Reference proteomes can be downloaded from https://www.uniprot.org/proteomes; manually annotated contaminants, or the amino acid sequences of the fusion construct, including the biotin ligase, can be included in the file if necessary

6. Proteomics raw data, lists of samples, and MaxQuant parameter (mqpar.xml) files associated with this protocol are deposited in PRIDE, https://www.ebi.ac.uk/pride/archive?keyword=baubec, under the accession numbers PXD014483 and PXD034918 or BioStudies, www.ebi.ac.uk/biostudies, under the accession number S-SCDT-10_1038-S44318-026-00768-2; relevant scripts can be found at https://github.com/BaubecLab/Setd2/tree/main/chromID

## Procedure

*Notes:*

*1. Generation of cell lines stably expressing eCRs fused to BASU or TurboID: Although this protocol was established with the parbit-v9 RMCE backbone, alternative expression vectors and fusion designs can be used, provided that the resulting fusion proteins retain efficient proximity-labeling activity. Cloning and generation of stable cells expressing the eCRs and the corresponding nuclear control can be performed as previously described [4–6]. eCRs used to generate stable cell lines are described in the Supplementary Table 1.*

**Figure 1.**
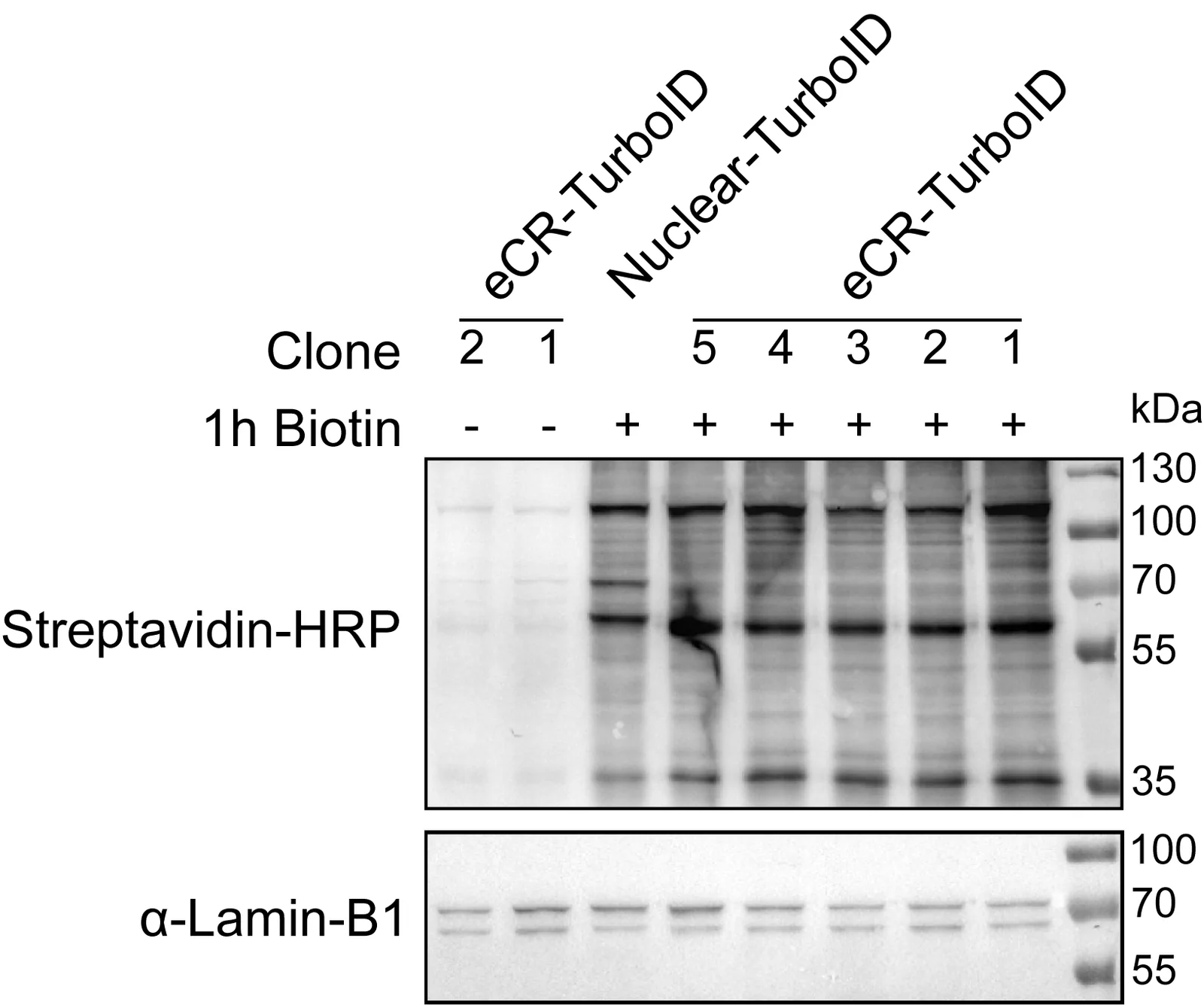
Validation of TurboID-mediated biotinylation in nuclear extracts by western blot. Nuclear extracts from representative clonal cell lines stably expressing a single MBD domain from the protein MBD1 fused to TurboID (eCR-TurboID) or Nuclear-TurboID were analyzed by western blot following incubation in the absence (-) or presence (+) of D-biotin for 1 h. Biotinylated proteins were detected using streptavidin–HRP. Alternatively, proteins can be detected with Streptavidin-Alexa 790. Cells incubated without D-biotin served as a negative control and are expected to show minimal background signal, whereas D-biotin-treated cells display robust biotinylation of proximal proteins. α-Lamin-B1 was used as a loading control. Biotinylation patterns and signal intensities may vary depending on the selected eCR and the level of TurboID fusion protein expression.

*2. Validation of stable cell lines: Validate fusion protein expression and biotinylation activity by western blot using labeled streptavidin or antibodies against biotin, as previously described [4–6]. Successful integration and expression of the TurboID (or BASU) construct should result in a strong streptavidin signal following D-biotin treatment, whereas untreated cells should display minimal background (Figure 1). Biotinylation efficiency and signal intensity may vary depending on the selected eCR and labeling conditions. Once validation is complete, proceed with the protocol described below.*

**A. Cell culture and proximity biotin labeling**

**Day 1**

1. Seed cells stably expressing the selected eCR fused to TurboID or BASU and the corresponding NLS-TurboID/BASU control onto 15-cm culture dishes for each biological replicate (we recommend n = 4 per construct/condition). See General notes 1 and 2.

2. Culture cells under standard growth conditions [4,12].

*Note: Seed mouse embryonic stem cells (mESCs) at ~4 × 10^6^ cells per dish (serum-LIF) and human osteosarcoma (U2OS) cells at ~7 × 10^6^ cells per dish.*

**Day 2**

3. Replace culture medium 24 h before cell harvesting when necessary.

*Notes:*

*1. Under such conditions, cells should reach approximately 85%–95% confluency and approximately 20–25 × 10^6^ cells (mESCs) or 20–25 × 10^6^ cells (U2OS cells) per dish at day 3 (~48 h post seeding).*

*2. Expected cell yield per dish is cell line and condition-dependent, reflecting differences in doubling time and confluent (saturation) density. We recommend verifying both parameters for your own cell line.*

*3. Because day 2 is experimentally demanding, plan cell seeding and culture time accordingly.*

**Day 3**

1. Supplement each 15-cm dish containing 20 mL of culture medium with 20 μL of a 50 mM D-biotin stock solution to achieve a final concentration of 50 μM.

2. Incubate cells for 1 h at 37 °C to induce proximity-dependent biotin labeling. See General note 3.

3. Immediately proceed to nuclei isolation and nuclear extract preparation after labeling.

**B. Cell harvesting and nuclei isolation**

**Day 3 (continued)**

1. Remove culture medium and wash cells once with 20 mL of ice-cold (D)PBS.

2. Remove (D)PBS, add 10 mL of ice-cold (D)PBS to each dish, and detach cells by scraping.

3. Transfer cell suspensions to 50-mL conical tubes. See General note 4.

4. Centrifuge samples at 120× *g* for 7 min at 4 °C.

5. Remove the supernatant completely and keep the cell pellets on ice.

**Caution:** Do not freeze the cell pellets after harvesting, as freeze thaw–induced lysis may affect protein recovery and increase nonspecific protein contamination in the final nuclear extract.

*Note: All subsequent steps are carried out on ice or at 4 °C unless otherwise indicated.*

6. Hypotonic swelling and nuclei isolation

a. Resuspend each cell pellet by gently swirling the conical tube in 1.5 mL of ice-cold NEB1. See General note 5.

**Critical:** Inadequate swelling leads to poor nuclei release.

b. Incubate samples on ice for 10 min to allow hypotonic swelling.

c. Centrifuge samples at 2,000× *g* for 10 min at 4 °C.

d. Carefully remove the supernatant.

e. Gently resuspend the pellet in 400 μL of ice-cold NEB1.

f. Transfer the suspension to a pre-chilled Dounce homogenizer.

**Caution:** Pipette slowly as the suspension may be highly viscous. See General note 6.

g. Homogenize samples by giving 10 strokes with the loose-fitting pestle (Pestle A), avoiding foaming.

h. Transfer homogenized samples to pre-chilled 1.5 mL tubes.

**Caution:** Do not vortex or pipette samples during hypotonic swelling steps, as this may disrupt nuclear integrity.

i. Centrifuge samples at 2,000× *g* for 10 min at 4 °C.

j. Remove the supernatant (this is the cytoplasmic extract) and snap-freeze in liquid nitrogen, if needed for quality control by western blot.

*Notes:*

*1. The extent of visible hypotonic swelling may vary substantially between cell types. mESCs typically display pronounced swelling, whereas more mechanically robust cell lines, such as U2OS, may exhibit only subtle changes before homogenization.*

*2. An additional wash step with 400 μL of ice-cold NEB1 is recommended for U2OS cells to minimize potential contaminations with cytoplasmic proteins.*

*3. The resulting nuclei pellet should correspond to approximately half of the initial cell pellet volume.*

**C. Nuclear extract preparation**

1. Resuspend isolated nuclei in 200 μL of NEB1 supplemented with Benzonase (4 μL of 25 U/μL Benzonase per sample).

2. Incubate samples for 2 h at 4 °C on a rotating wheel.

**Critical:** Complete Benzonase digestion yields a homogeneous nuclear extract, whereas incomplete digestion is characterized by persistent viscosity and the presence of readily visible aggregates. If incomplete digestion is observed, continue the Benzonase incubation for an additional 1 h before proceeding.

3. Centrifuge samples at 2,000× *g* for 10 min at 4 °C.

4. Remove the supernatant and resuspend nuclei in 187.5 μL of NEB2-450. See General note 4.

5. Transfer suspensions to a pre-chilled Dounce homogenizer.

6. Homogenize nuclei using 10 strokes with the tight-fitting pestle (Pestle B).

7. Transfer samples to pre-chilled 1.5 mL Safe-Lock tubes.

8. Vortex for 15 s and incubate samples on a rotating wheel for 1 h at 4 °C.

9. Vortex samples again after incubation.

10. Centrifuge samples at 16,000× *g* for 15 min at 4 °C.

11. Transfer the supernatant (this is the nuclear extract) to fresh pre-chilled low protein binding tubes.

**Pause point:** Nuclear extracts can either be snap-frozen in liquid nitrogen and stored at -80 °C or directly processed for streptavidin pull-downs.

**D. Streptavidin affinity purification**

**Day 4**

**D1. Preparation of streptavidin beads**

1. Transfer 10 μL of streptavidin Sepharose high-performance beads per pull-down reaction to 1.5 mL tubes. See General note 7.

2. Wash beads twice with 1 mL of RIPA buffer.

3. During each wash, invert tubes 10 times and centrifuge at 1,000× *g* for 2 min at 4 °C.

4. Resuspend beads in 92 μL of RIPA buffer per reaction.

5. Distribute 95 μL of bead suspension into individual tubes.

6. Centrifuge samples again at 1,000× *g* for 2 min at 4 °C and discard the supernatant.

**D2. Affinity purification of biotinylated proteins**

1. Lower the NaCl concentration of the nuclear extracts from 450 to 150 mM by dropwise addition of ice-cold NEB2-NS using a volume equal to twice the volume of the nuclear extract.

**Caution:** Dropwise addition prevents local protein precipitation.

2. Centrifuge extracts at 16,000× *g* for 15 min at 4 °C.

3. Transfer clarified nuclear extracts to fresh pre-chilled 2-mL tubes.

4. Quantify protein concentration using Qubit or an equivalent protein quantification assay.

**Critical:** Equal protein concentrations between samples are essential for comparative proteomic analyses.

5. Adjust samples to 0.3% IGEPAL with 10% IGEPAL solution.

6. Normalize the volumes of all samples with IPB if necessary. See General note 4.

7. Add nuclear extracts to tubes already containing streptavidin beads.

8. Incubate samples overnight (no more than 18 h) at 4 °C on a rotating wheel.

**Critical:** Over-incubation increases nonspecific background.

*Note: Nuclear extraction typically yields approximately 200–300 μg of protein per 15-cm dish. We recommend scaling the concentration of nuclear extract for the highest concentration possible to maximize recovery of biotinylated proteins.*

**E. High-stringency washing of streptavidin beads**

**Day 5**

1. Wash beads twice with 1 mL of 2% SDS in TE (+DTT and +PIC) at RT.

2. Wash beads once with 1 mL of ice-cold HSB (+DTT and +PIC).

3. Wash beads once with 1 mL of ice-cold DOC buffer (+DTT and +PIC).

4. Wash beads four times with 1 mL of ice-cold TE (no DTT and no PIC).

5. After the final wash, carefully remove residual liquid using a 26G needle.

**Caution:** Do not allow beads to dry between wash steps.

*Notes:*

*1. The SDS washing step can be replaced with two additional washes with HSB to reduce stringency.*

*2. Perform all washes by inverting tubes 10 times followed by centrifugation at 1,000× g for 2 min at 4 °C.*

*3. Specifically, during washing step 1, keep samples at RT to prevent SDS precipitation.*

**Critical:** Excessive carryover of detergents can inhibit trypsin activity and interfere with downstream LC-MS analysis; TE washes at step 4 are therefore critical.

*4. Wash stringency may require optimization depending on the bait protein and labeling background.*

**F. On-bead tryptic digestion and peptide recovery**

**F1. Reduction and alkylation**

1. Resuspend streptavidin beads in 50 μL of freshly prepared urea elution buffer.

2. Incubate samples for 20 min at RT in a thermomixer at 1,400 rpm.

3. Add chloroacetamide to a final concentration of 50 mM.

**Caution:** Chloroacetamide is light-sensitive; keep it in the dark for effective alkylation.

4. Incubate samples for 10 min at RT in the dark.

**F2. On-bead digestion**

1. Add 2.5 μL of trypsin (0.1 μg/μL) to the solution.

2. Incubate samples for 2 h at RT at 1,400 rpm in the dark.

3. Centrifuge samples at 1,500× *g* for 2 min at RT and transfer the supernatant to a fresh tube.

4. Add an additional 50 μL of urea elution buffer to the beads.

5. Incubate samples for 5 min at RT at 1,400 rpm.

6. Centrifuge again and combine both supernatants.

7. Add 1 μL of trypsin (0.1 μg/μL) to the pooled supernatants, vortex, and spin down shortly.

8. Incubate samples overnight at RT without agitation to perform in-solution tryptic digestion.

**F3. Peptide recovery**

**Day 6**

1. Stop tryptic digestion by adding TFA to a final concentration of approximately 0.5% from the freshly prepared 10% TFA solution.

2. Verify that the sample pH is ≤3 (by pipetting 1 μL of a single selected sample to a pH strip).

3. Proceed immediately to peptide desalting.

**G. Peptide desalting using C18 Spin Tips**

**G1. Equilibration of C18 Spin Tips**

1. Prepare C18 Spin Tips by inserting them into centrifuge adaptors seated in 2-mL tubes or by placing them into 2-mL tubes with pinched lids.

2. Place the tubes with the Spin tip in the centrifuge and keep them there until completion of step G1.5 (carefully add solutions directly to the tubes and start centrifugations accordingly).

3. Activate tips with 50 μL of 100% methanol and centrifuge.

4. Wash tips with 50 μL of freshly prepared Spin Tip buffer B and centrifuge.

5. Equilibrate tips twice with 50 μL of freshly prepared Spin Tip buffer A and centrifuge.

*Notes:*

*1. Unless otherwise stated, centrifuge C18 Spin Tips at 1,000× g for 1 min.*

*2. In steps G1.1–4, discard the flowthrough if necessary to prevent contact between the Spin Tip and the collected liquid.*

*3. Alternatively, C18 Spin Columns can also be used according to the manufacturer’s instructions.*

**G2. Peptide binding and elution**

1. Load digested samples onto C18 Spin Tips mounted in new tubes.

2. Centrifuge at 1,000× *g* for 3 min at RT.

3. Reapply the flowthrough to its corresponding Spin Tips.

4. Centrifuge samples at 1,000× *g* for 3 min at RT.

5. Wash Spin Tips once with 50 μL of Spin Tip buffer A and centrifuge at 1,000× *g* for 1 min at RT.

6. Elute the samples into new 1.5-mL low protein binding tubes using 2× 30 μL of Spin Tip buffer B and centrifuge at 1,000× *g* for 1 min at RT.

7. SpeedVac samples at RT to near dryness.

8. Store peptides at -80 °C or resuspend peptides in an LC-MS/MS compatible buffer. Samples are now ready for LC-MS/MS analysis. For more details regarding LC-MS/MS setup, see [5] and General note 8.

**Pause point:** C18 Spin Tips may be stored at 4 °C before peptide elution. Before storage, add 50 μL of Spin Tip buffer A and centrifuge at 500× *g* for 15 s to remove excess liquid while maintaining the membrane hydrated.

## Data analysis

This section requires basic familiarity with proteomics software (MaxQuant, Perseus) and working knowledge of R for quality control and visualization. No command-line/Linux expertise is required. R can be installed locally. For examples of data analysis workflows, threshold selection, and details for data analyses (points 1–5 below), see [4,5].

A full description of the acquisition and analysis parameters used in the original studies, including the complete MaxQuant configuration, is provided in the deposited mqpar.xml parameter file accompanying the proteomics raw data.

Recommended replication: Process at least three, and preferably four, independent biological replicates per construct/condition, each analyzed against the matched nuclear-localized TurboID/BASU (lacking the reader domain) as control. The statistical workflow below is designed for triplicate or quadruplicate comparisons.

**1. Protein identification and label-free quantification:** Process the LC-MS/MS raw files in MaxQuant using the integrated Andromeda search engine for protein identification and label-free quantification (LFQ). Search against the appropriate reference proteome. The configurations used in MaxQuant for protein identification can be found in the deposited mqpar.xml file, PXD014483, at https://www.ebi.ac.uk/pride/archive/projects/PXD014483. Different versions of MaxQuant can be used if users verify that the relevant functions, parameters, and default settings have not changed between versions.

**2. Quality control:** Assess LC-MS run quality with PTXQC (https://github.com/cbielow/PTXQC) in R, using the MaxQuant output as input.

**3. Filtering and normalization:** Filtering and normalization of protein hits are performed using Perseus. Different versions of Perseus can be used; however, users should verify that the relevant functions, parameters, and default settings have not changed between versions.

**4. Statistical enrichment:** Identify enriched proteins using a two-tailed, two-sample t-test comparing the eCR with the NLS-only control. Determine significance thresholds according to experimental conditions, fusion proteins, and the scientific question being addressed. Significant proteins can be selected using log2 fold enrichment and p-value cutoffs or by applying permutation-based FDR thresholds.

**5. Visualization:** Export the results and visualize enrichment using R as volcano plots of log2 fold enrichment (fold change) versus -log_10_ p-value, with significant proteins highlighted either by conventional significance cutoffs or by FDR-based significance threshold curves incorporating an S_0_ parameter.

## Validation of protocol

This protocol has been used and validated in the following open-access research articles:

• Villaseñor et al. [4]. ChromID identifies the protein interactome at chromatin marks. *Nature Biotechnology* (Figure 3a–e and Figure 5a, c, d, e).

• Cardoso da Silva et al. [5]. Probing DNA damage sites reveals context-dependent and novel DNA damage response factors. *bioRxiv* (Figure 4a, c, d–j and Figure 5a–h).

• Ambrosi et al. [6]. The H3K36me3 methyltransferase SETD2 contributes to PAF1C interactions with RNA Pol II and is required for neuronal differentiation. *The EMBO Journal* (Figure 5a–e).

• Butz et al. [7]. DNA sequence and chromatin modifiers cooperate to confer epigenetic bistability at imprinting control regions. *Nature Genetics* (Figure 5c).

## General notes and troubleshooting

**General notes**

1. Include cells stably expressing nuclear-localized TurboID/BASU as background controls.

2. This protocol has been optimized and validated in mESCs and the human osteosarcoma cell line U2OS. Only minor adjustments were required to accommodate the specific growth characteristics of each cell line. This suggests that the protocol is readily adaptable to other cell types with minimal optimization. Direct comparisons between different cell lines should be interpreted with caution because differences in chromatin organization, protein expression, and labeling efficiency may influence the results. We therefore recommend comparing conditions within the same cell line using the corresponding matched nuclear-localized TurboID/BASU control.

3. Optimize biotin concentration and labeling duration based on the cell type, ligase activity, and fusion protein expression level. Before quantitative analyses, validate fusion protein expression, chromatin localization, and biotinylation efficiency by western blot and/or immunofluorescence via labeled streptavidin.

4. Replicates may be pooled to reduce sample handling variability during nuclei isolation and extraction. Pooling can be performed from step B3 (day 3) through step D2.6 (day 4). When pooling samples, adjust all reagent volumes proportionally to the total number of replicates combined. Importantly, the pooled sample should be divided back into individual replicates before the streptavidin pull-down step (step D2.6, day 4), which should be performed separately for each individual replicate.

5. All volumes are optimized for the number of cells listed in the protocol (~85%–95% confluency in a 15-cm dish). For different cell lines and cell numbers, scale volumes accordingly. As a reference, estimate cell pellet volume (PV) (by comparing to water). For NEB1, add 5 PV of ice-cold NEB1. Adjust all subsequent volumes and concentrations proportionally using this volume as a reference until section C.

6. It is advised to use non-autoclaved tips and centrifuge tubes to prevent microplastics from entering the LC-MS/MS, as autoclaving may increase polymer release and contamination. Additionally, use standard filter tips, clean consumables, and fresh Milli-Q water when preparing solutions that are filter-sterilized to minimize contamination.

7. This protocol recommends the use of Streptavidin Sepharose high-performance beads for streptavidin pull-down. As an alternative, Dynabeads M-280 Streptavidin beads (Thermo Fisher) can also be used; however, in our hands, these beads resulted in increased background signal compared to Streptavidin Sepharose high-performance beads.

8. Peptides purified with this protocol were analyzed by data-dependent acquisition (DDA), and preliminary tests using data-independent acquisition (DIA) also produced high-quality datasets. We recommend optimizing the acquisition method for your specific samples.

9. For peptide desalting using C18 Spin Tips, prepare all solutions fresh immediately before use, preferably in a glass container. Avoid preparing or storing MS-grade solutions in Falcon tubes or other plastic containers, as polymers may introduce contaminants that interfere with LC-MS/MS analyses.

**Troubleshooting**

**Table:** 

Problem	Cause	Solution
Low or absent biotinylation signals detected by western blot (WB) and/or immunofluorescence (IF) with anti-biotin	Insufficient/excessive biotin concentration or labeling time.	Verify if biotin stock is freshly dissolved in DPBS and not degraded. Shorten/titrate the labeling time.
	Biotin ligase (BASU/TurboID) is poorly expressed or unstable in the cell line.	Verify the expression of the fusion protein by WB against the fused protein before proceeding to pull down.
	The fusion protein fails to localize to the nucleus or target chromatin compartment.	Confirm (sub)nuclear localization by immunofluorescence with anti-biotin combined with antibodies to the selected target protein. Check whether the fusion protein is functional.
	Biotin ligase activity is compromised by fusion orientation or steric hindrance.	Test N- vs. C-terminal ligase fusions and make sure you include a flexible linker in the constructs with the biotin ligase.
	Short-lived or weak chromatin interactions are insufficiently labeled.	Increase labeling duration within non-toxic limits and validate labeling kinetics experimentally. We have obtained good results with 12-h labeling. Even for TurboID, 12-h labeling is possible, depending on the selected protein's affinity for the target.
Excessive nonspecific biotinylation/background labeling	Excessive biotin ligase expression or prolonged labeling.	Reduce labeling time or expression levels; use inducible systems if necessary.
	Endogenous biotin-containing proteins dominate the signal.	Under our experimental conditions, biotin-depleted media were not necessary; however, they may be considered if endogenous protein biotinylation is excessively high. We recommend assessing background labeling by comparing WB signals between the cell line expressing the fusion protein and wild-type cells in the presence and absence of biotin addition.
Insufficient pelleting of cells and the presence of floating cells in the supernatant	Too many cells were harvested, or the characteristics of cells differ substantially from those tested in this protocol (mESCs and U2OS cells).	Increase the duration of centrifugation.
Cell swelling is subtle or barely visible after incubation with NEB1 buffer	The volume of the NEB1 buffer is insufficient.	Adjust the volume of the NEB1 buffer by estimating pellet volumes (see General note 5). Mouse embryonic stem cells typically display pronounced swelling following incubation in NEB1, whereas more mechanically robust adherent cell lines, such as U2OS, may exhibit only subtle morphological changes before homogenization. In these cases, check for successful nuclei isolation only after Dounce homogenization, rather than visible swelling alone.
High proportion of missed cleavages or incomplete digestion	Insufficient trypsin activity.	Increase the digestion time or the amount of trypsin.
Poor reproducibility between biological replicates	Variability in cell growth, labeling efficiency, or extraction conditions.	Standardize culture conditions, confluency, labeling duration, and extraction workflow.
Low recovery of chromatin-associated or bait-proximal proteins	Inefficient biotin labeling; weak bait expression/localization.	Validate bait expression and chromatin localization; optimize labeling duration and biotin concentration.
Excessive streptavidin-derived peptides or bead contaminations	Bead carryover into peptide digest.	Perform a double centrifugation step to remove excess beads.

## Supplementary information

The following supporting information can be downloaded here:

1. Supplementary Table 1. List of engineered chromatin readers (eCRs) used for ChromID.
